# Responsiveness of the cervical joint position error test to detect changes in neck proprioception following four weeks of home-based proprioceptive training

**DOI:** 10.1371/journal.pone.0303066

**Published:** 2024-05-10

**Authors:** Ahmad AlDahas, Valter Devecchi, Janet A. Deane, Deborah Falla

**Affiliations:** 1 Centre of Precision Rehabilitation for Spinal Pain (CPR Spine), School of Sport, Exercise and Rehabilitation Sciences, College of Life and Environmental Sciences, University of Birmingham, Birmingham, United Kingdom; 2 Department of Physical Therapy, Al-Sabah Medical Hospital, Ministry of Public Health, Shuwaikh Industrial, Kuwait; King Khalid University, SAUDI ARABIA

## Abstract

**Introduction:**

People with chronic neck pain (CNP) commonly exhibit a range of physical impairments including cervical proprioceptive deficits. Assessing proprioception using a head mounted laser to assess joint position error (JPE) is a reliable and valid measure. However, the responsiveness of this measure has not been assessed.

**Objective:**

To assess the responsiveness of the measure of cervical JPE after a 4-week home-based neck proprioceptive training intervention in people with CNP.

**Design:**

An observational study to assess the responsiveness of the measure of cervical JPE.

**Methods:**

The JPE test was assessed in people with CNP before and after 4 weeks of neck proprioception training. JPE was assessed as participants performed neck joint position sense tests for flexion, extension, right rotation, and left rotation in sitting and standing which were performed in a random order. Both the absolute and constant JPE were assessed. The intervention consisted of neck repositioning exercises as well as movement sense exercises. Cohen’s d effect size was used to assess the internal responsiveness of the JPE test. The Pearson’s correlation was used to assess the change of scores of the laser pointer and measures from inertial measurement units (IMUs) (external responsiveness).

**Results:**

After 4 weeks of proprioception training, JPE assessed in sitting reduced from 2.69^**◦**^-3.57^**◦**^ to 1.88^**◦**^-1.98^**◦**^ for flexion, extension, and right rotation with large effect sizes (Cohen’s d range: 1.25–2.00). For left rotation, JPE reduced from 3.23^**◦**^ to 1.9^**◦**^, and the effect size was close to being large (Cohen’s d: 0.79). When assessed in standing, JPE reduced from 3.49^**◦**^-4.52^**◦**^ to 1.5^**◦**^-2.33^**◦**^ with large effect sizes (Cohen’s d range: 0.89–1.25) for flexion, extension, right rotation, and left rotation. Large effect sizes were not observed for the constant JPE when assessed in either sitting or standing. The assessment of the external responsiveness revealed weak correlations between the change of scores obtained from the laser pointer and the IMUs for all movements, apart from the constant JPE in sitting for left rotation, which showed a strong correlation (r = 0.7).

**Conclusion:**

The results of this study showed that the measure of the JPE has sufficient internal responsiveness, however, the external responsiveness was inadequate. Further research is advised.

## Background

Neck pain is a common musculoskeletal condition worldwide, causing substantial disability and economic burden [1]. It is one of the most common musculoskeletal conditions with an annual prevalence ranging from 12% to 71% [2]. Although most people with neck pain have a good prognosis, one-third will go on to develop chronic neck pain (CNP) [3]. Beside pain, several physical impairments are commonly present in people with CNP such as altered neuromuscular control [4–7], reduced range of motion (ROM) and smoothness of movement [8–12], and impaired head-eye movement control [11]. A further common feature in people with CNP is reduced proprioceptive acuity of the neck, especially for those presenting with whiplash associated disorders (WAD) [13,14] and the symptom of dizziness [15].

Cervicocephalic kinaesthesia is the ability to perceive both movement and the location of the head in space and in relation to the trunk and is therefore vital for normal function [16–18]. Cervical kinesthesia is vitally dependent on sensory input from the, neck muscles (especially in the sub-occipital muscles), ligaments, and joints [18]. In a clinical environment, cervical kinesthesia can be assessed by evaluating head repositioning accuracy (HRA) and measuring joint position error (JPE). JPE can be used to measure the ability of the individual to reposition their head back to its neutral head position (NHP) or to a predefined target head position (THP) [19]. Revel et al. [20] was the first to describe the assessment of neck proprioception (i.e., cervicocephalic kinesthesia) using a laser pointer mounted on the head and revealed differences in proprioceptive acuity between people with and without CNP after returning from neck flexion, extension, and right and left rotation. Further work followed and there is now clear evidence from a systematic review of the literature that neck proprioception can be impaired in people with CNP, specifically WAD [21], especially those with the symptom of dizziness [21].

Randomised controlled trials (RCTs) have shown that neck proprioception training is beneficial for people with CNP and can reduce proprioceptive deficits. For example, Revel et al. [22] combined oculomotor training with proprioception training (head repositioning tasks) over a period of ten weeks and demonstrated that neck repositioning accuracy and ROM improved significantly when compared to a control group. Jull et al. [12] compared the effect of six weeks of proprioception exercises versus craniocervical flexion exercise and showed that both programs led to improvements in repositioning sense. However, participants allocated to the proprioception training group showed greater improvements compared to the craniocervical flexion training group when HRA was assessed for right rotation. Moreover, in a double-blinded RCT by Saadat et al. [23], it was found that proprioception training combined with traditional physical therapy interventions was more effective than traditional physical therapy treatment alone in improving cervical JPE.

For an outcome measure to be used in clinical settings, it must be affordable, safe, simple to administer, and able to be used within an operational time frame [24]. Moreover, outcome measures need to be reliable, valid, and responsive to detect a change [24]. The use of a laser pointer mounted on the participant’s head to assess cervical proprioception is reliable and valid [25]. Responsiveness of the measure, which is the ability of an outcome measure to detect a change over time [26], has not been examined before. In order to assess the effectiveness of a particular intervention, establishing the responsiveness of clinical tests used to evaluate outcomes is crucial [24]. Therefore, the aim of this study was to assess the internal and external (relative to measures obtained from inertial measurement units, IMUs) responsiveness of the cervical JPE test measured in people with CNP using a laser pointer and a wall target. We hypothesised that the measure of cervical JPE would demonstrate sufficient levels of responsiveness which would support its use in the clinical assessment of neck proprioception.

## Methodology

### Study design

This is a responsiveness study of the measure of cervical JPE following a period of 4-weeks of neck proprioception training for people with CNP. The COnsensus-based Standards for the selection of health status Measurement INstruments (COSMIN) guidelines of study designing checklist [27] was used to report this study. Before and after the intervention, testing was carried out by a single rater (AA) who was aware of the COSMIN guidelines for reporting studies of measurement properties and has received extensive training on data collection and analysis of cervical JPE. The study was carried out in a motion analysis laboratory at the Centre of Precision Rehabilitation for Spinal Pain (CPR Spine), University of Birmingham, United Kingdom from September to December 2022. The study received full ethical approval from the ethics committee at the University of Birmingham (ERN_22–0269). All participants provided written informed consent.

### Participants

Participants were recruited from the staff and student population at the University of Birmingham. Participants were initially screened via email through a health screening questionnaire to insure their eligibility for the study. G*Power (version 3.1.9.4) was used to determine the required sample size for this study. The sample size was determined based on a 0.05 significance level, a moderate to high effect size, and a power of 80%. Therefore, a sample size of seventeen participants was required.

#### Inclusion and exclusion criteria

Participants had to be aged 18–55 years to limit potential degenerative changes of the spine with older age [28] and presenting with neck pain for at least 3 months [29] with at least mild pain (≥4 out of 10) on a Numerical Rating Scale (NRS) [30] or at least 5 out of 50 on the Neck Disability Index (NDI) [29]. The NRS is a reliable and valid self-administered questionnaire to measure pain intensity [31]. The NDI is a reliable and valid method to measure perceived disability in people with CNP [32]. The exclusion criteria included history of neck trauma, neck surgery, vestibular disorders, and Covid-19 related symptoms in the last 14 days.

### Testing procedure

At the beginning of the first session, participants were asked to complete the Dizziness Handicap Inventory (DHI) and Tampa Scale of Kinesiophobia (TSK). The DHI is a questionnaire that is sub-grouped into three domains (functional, emotional, and physical) that assesses dizziness over 25 items [33]. The DHI scores range from 0 to 100 [34]; DHI scores can be further subdivided according to the sub-groups of the questionnaire into 28 points for physical, 36 points for functional, and 36 points for emotional [35]. The TSK contains 17 items that assess the person’s fear of movement and movement-related behaviour [36]; scores range from 17–68, where higher scores mean higher degrees of Kinesiophobia [36]. Pre and post training participants carried out the NHP task in sitting and standing with 15-minute rest intervals. Testing in sitting took place before standing and the order of tested movements was randomised using the following website (https://www.random.org/lists/). Active neck movements tested included flexion, extension, right rotation, and left rotation.

**Fig 1:**
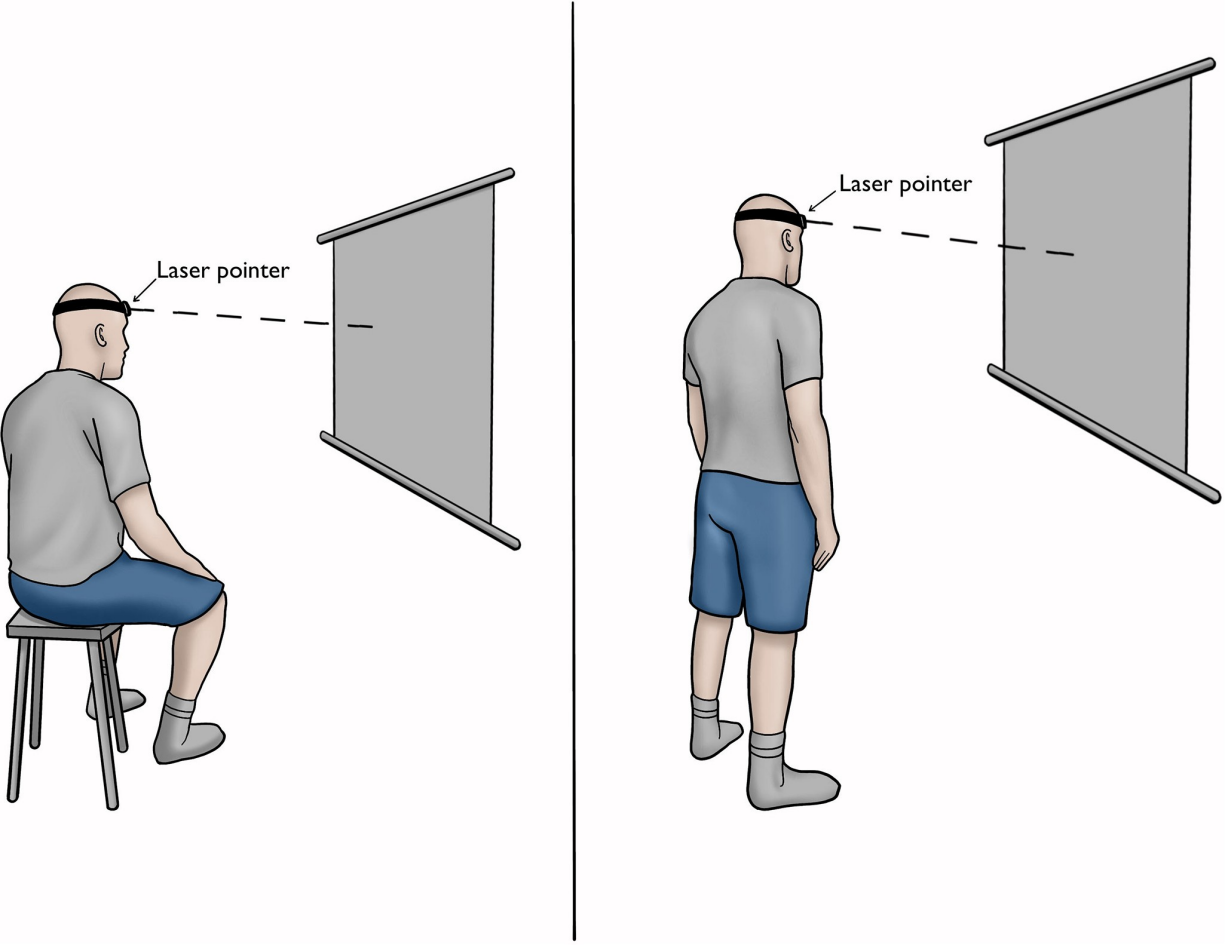
Measuring proprioception using a laser pointer mounted on the participant’s forehead. Data collection carried out in sitting and standing.

A laser pointer specifically designed for clinical testing of proprioception (https://www.concussionlab.com) was fixed on the participant’s forehead. The participant sat/stood in a comfortable position in front of an A2-sized paper, positioned 90cm in front of them at eye level (Fig 1). A test of repositioning to a NHP was then carried out. Participants were asked to stay in a neutral position for few seconds then the rater marked this position on the wall target as the starting position (i.e., NHP). The participant moved their head away from the start position to full range and then attempted to reposition the head back to the starting point as accurately as possible. The speed of their movements was not controlled, and they were simply asked to move at a comfortable pace.

Participants performed one familiarisation trial with their eyes open and subsequently, they were asked to close their eyes, perform each active neck movement and to repeat each movement six times, since six trials have been recommended to assess spinal proprioception [17,37]. Participants were instructed to keep their eyes closed across the full six repetitions. A one-minute rest was given between each movement direction. JPE was evaluated in the primary plane of movement only. The difference between the starting position (zero) and the returning point in the plane of movement was measured in centimetres [29,38,39] and then converted to degrees using this formula angle = tan^-1^ [error distance/90 cm] [39]. The average of the six trials was calculated and taken forward for data analysis. Both absolute and constant errors were determined. Absolute error (AE) is the mean of total deviation from the target ignoring the positive and negative values [40] whereas constant error (CE) is the mean of total deviation from the target considering positive and negative values [40].

After that, participants rested for 15 minutes before repeating the same procedure but with measurements taken from IMUs (Noraxon Research PRO IMU, Noraxon, USA) with a sampling rate of 100 Hz. [41]. One IMU sensor was fitted on the forehead via double-sided tape and one sensor was fitted over the neck at the level of the seventh cervical spinous process. Prior to data collection, the IMUs were calibrated, and the starting position was set at 0°. The IMUs by Noraxon are reliable and valid for measuring body kinematics [41]. To analyse the IMU data, the two waveforms of neck flexion/extension and right rotation/left rotation were exported from myoRESEARCH software (Noraxon, USA) to MATLAB where they were analysed with customised script. The waveform referring to the primary plane of movement was considered. The starting position was visually identified from the angle and velocity waveforms, and it was recognised by the steady position just before starting the movement. The return position was considered as the one at the end of the recording. The JPE was then computed as the difference between the start and return position in degrees. Both absolute and constant JPE were computed.

### Proprioception training

After the first testing session, participants were instructed on how to conduct four exercises and they were given a laser pointer for home use. Participants were instructed to perform the exercises for a period of four weeks and each exercise was to be carried out twice a day, three times a week. The specific duration of training was chosen as four weeks as this duration of proprioception training has been shown to be effective in previous research [18]. Each exercise was carried out in both sitting and standing. An investigator followed up with the participants once a week via email. This was to ensure that their training was running smoothly without adverse effects or symptoms. After four weeks of training, the participants were asked to attend a follow-up measurement session the following day or as early as possible (not greater than five days). The same testing procedure was carried out as in the first session.

The training intervention consisted of head repositioning exercises [22] as well as movement sense exercises [42,43]. For the head repositioning exercises, the participants performed the NHP task for the first two weeks then progressed to THP tasks afterwards. The movement sense exercises consisted of three exercises: tracing lines of ZZ bands, different shaped circles, and different arrow patterns. For the different circle sizes exercise, participants progressed in training from tracing bigger circles to smaller ones. A detailed description of the exercises can be found in S1 File.

### Statistical analysis

Data analysis was carried out using SPSS (version 29.0.1, IBM). As recommended by COSMIN [44], the effect size (Cohen’s d) of pre-post differences was used to measure the internal responsiveness of the measure of JPE using the formula Cohen’s d = (M1-M2)/SDpooled, where M1 is the mean of baseline measurements, M2 is the mean of follow-up measurements, and SDpooled is the standard deviation of the two measurements [45,46]. For interpretation, d = 0.2 is considered a small effect, d = 0.5 is a medium effect, and 0.8 is a large effect [45,46]. A further sub-analysis was carried out only for participants that had an absolute JPE of ≥3^◦^ since an error of ≥3^◦^ to 4^◦^ can indicate a proprioceptive deficit [47]. The Pearson’s product-moment correlation coefficient (r) was used to analyse the external responsiveness (correlation of change in scores of the laser pointer and the IMUs) of the measure of JPE for each movement. The following criteria was used for interpretation; 0.1–0.39 = weak, 0.4–0.69 = moderate, 0.7–0.89 = strong, 0.9–1 = very strong [48]. The Shapiro-Wilk test was used to assess the normality of the distribution of the questionnaires. Since the data were not normally distributed, the Wilcoxon Signed Rank Test was carried out to investigate changes in pain intensity (NRS), disability (NDI), fear of movement (TSK), and dizziness (DHI), and repositioning error before and after the intervention. The level of significance (P-value) was set at 0.05.

## Results

Nineteen participants (8 males, 11 females) with CNP took part in this study with an age (mean and standard deviation (SD)) of 27.73 (6.20) years, height of 172.21 (9.40) cm, and weight of 73.72 (15.76) kg. After 4 weeks of proprioception training, significant reductions in NDI, NRS, and TSK scores were observed. There was no significant change on the DHI (Table 1). The minimal clinically important change for the NDI is a decrease of 10.5 points and for the NRS is a decrease of 4.3 points [49]. In the current study, the NDI post intervention decreased by 10.86 points, which is clinically significant. The change in pain intensity based on the NRS however, did not reach a minimal clinically important change. Regarding the TSK, a decrease of 4 points is considered clinically significant [50] and this was not obtained post intervention.

**Table 1:** Mean, standard deviation, and range of the scores for patient reported outcome measures assessed at baseline and after the four-week training intervention.

Questionnaire (score range)	Baseline (Mean, (SD), Range)	Follow-up (Mean, (SD), Range)	P-value
**NDI (0–100)**	28.75 (8.75), 18–50	17.89 (8.01), 4–32	0.001*
**NRS (0–10)**	4.7 (1.7), 2–7	3.39 (1.99), 0.5–7	0.01*
**TSK (17–68)**	36.6 (5.51), 30–48	34.73 (6.85), 24–49	0.02*
**DHI (0–100)**	20.73 (9.38), 6–38	19.26 (9.75), 0–36	0.45

NDI = Neck Disability Index. NRS = Numerical Rating Scale. TSK = Tampa Scale of Kinesiophobia. DHI = Dizziness Handicap Inventory.

* = statistically significant (P-value ≤0.05).

When assessed in sitting, after 4 weeks of proprioception training, the absolute JPE significantly reduced in all of the movements tested, apart from right rotation which was not significant (P = 0.07) (Table 2). Similar changes were also seen for all movements when the absolute JPE was assessed in standing (Table 3). In contrast, the constant JPE did not change regardless of whether it was assessed either in sitting or standing (Tables 2 and 3). When using the laser pointer, the Cohen’s d effect size for the measure of absolute JPE assessed in sitting showed that there was a large effect following four weeks of proprioceptive training and this was evident for relocation from neck flexion, extension, right rotation, with the measure from left rotation approximating large effect size (Cohen’s d = 0.79) (Table 2). Large effect sizes were also found for absolute JPE when assessed in standing following active neck flexion, extension, right rotation, and left rotation (Table 3). In contrast, large effect sizes were not observed for the constant JPE when assessed in either sitting or standing (Tables 2 and 3). A sub-analysis established that for participants with an absolute JPE of ≥3◦. Following the proprioception training intervention, the absolute JPE, assessed in sitting, showed a significant reduction when assessed in flexion, extension, and left rotation, while right rotation was not significant (P = 0.27) (Table 4). When assessed in standing, a significant reduction in absolute JPE was seen for all movement directions (Table 4). Large effect sizes (Cohen’s d) were observed for the absolute JPE in all movement directions both in sitting and standing (Table 4).

**Table 2:** Mean and standard deviation of the absolute and constant JPE measured in sitting at baseline and at the follow-up session using a laser pointer. Cohen’s d effect sizes demonstrate the internal responsiveness of the measures.

Error type	Absolute error		P-value	Effect size (Cohen’s d)	Constant error		P-Value	Effect size (Cohen’s d)
JPE	Baseline Mean^◦^ (SD^◦^)	Follow-up Mean^◦^ (SD^◦^)			Baseline Mean^◦^ (SD^◦^)	Follow-up Mean^◦^ (SD^◦^)		
**Flexion**	3.57 (1.59)	1.98 (0.93)	0.003*	1.25	-0.33 (3.58)	-0.84 (1.7)	0.46	0.19
**Extension**	5.17 (2.54)	1.88 (0.75)	<0.001*	2.008	0.76 (5.31)	-0.04 (1.7)	0.35	0.23
**Right rotation**	2.69 (1.91)	1.91 (1.69)	0.07	2.75	-0.54 (3.23)	0.06 (2.31)	0.74	-0.22
**Left rotation**	3.23 (2.06)	1.9 (1.27)	0.01*	0.79	-0.3 (4.07)	-0.28 (2.04)	0.87	-0.005

JPE = joint position error.

* = statistically significant (P-value ≤0.05).

**Table 3:** Mean and standard deviation of the absolute and constant JPE measured in standing at baseline and at follow-up session using a laser pointer. Cohen’s d effect sizes demonstrate the internal responsiveness of the measures.

Error type	Absolute error		P-value	Effect size (Cohen’s d)	Constant error		P-value	Effect size (Cohen’s d)
JPE	Baseline Mean^◦^ (SD^◦^)	Follow-up Mean^◦^ (SD^◦^)			Baseline Mean^◦^ (SD^◦^)	Follow-up Mean^◦^ (SD^◦^)		
**Flexion**	4.52 (2.8)	2.1 (1.00)	0.002*	1.27	0.29 (5.13)	-1.18 (1.78)	0.17	0.43
**Extension**	3.96 (1.85)	2.33 (1.77)	0.009*	0.89	-0.67 (3.74)	0.63 (2.67)	0.13	-0.4
**Right rotation**	3.53 (1.7)	1.67 (1.31)	<0.001*	1.23	-0.35 (3.79)	0.42 (1.92)	0.39	-0.27
**Left rotation**	3.49 (2.49)	1.5 (0.67)	<0.001*	1.25	-1.05 (3.84)	-0.53 (1.12)	0.71	-0.2

JPE = joint position error.

* = statistically significant (P-value ≤0.05).

**Table 4:** Mean and standard deviation of the absolute JPE measured in sitting and standing at baseline and at follow-up session for a subgroup of participants with ≥3◦ of absolute JPE using a laser pointer. Cohen’s d effect sizes demonstrate the internal responsiveness of the measures.

Position	Sitting		P-value	Effect size (Cohen’s d)	Position	Standing		P-value	Effect size (Cohen’s d)
JPE	Baseline Mean^◦^ (SD^◦^)	Follow-up Mean^◦^ (SD^◦^)			JPE	Baseline Mean^◦^ (SD^◦^)	Follow-up Mean^◦^ (SD^◦^)		
**Flexion (n = 12)**	4.36 (1.48)	1.68 (0.45)	0.002*	2.75	**Flexion (n = 10)**	6.61 (2.35)	2.33 (1.22)	0.007*	2.39
**Extension (n = 15)**	5.94 (2.66)	1.64 (0.62)	<0.001*	2.6	**Extension (n = 12)**	4.85 (1.79)	2.61 (2.07)	0.02*	1.15
**Right rotation (n = 4)**	5.95 (1.62)	3.36 (3.07)	0.27	1.1	**Right rotation (n = 12)**	4.44 (1.45)	1.8 (1.53)	0.002*	1.76
**Left rotation (n = 9)**	4.62 (2.19)	1.71 (0.73)	0.008*	1.98	**Left rotation (n = 7)**	6.29 (2.01)	1.57 (0.69)	0.01*	3.48

JPE = joint position error. n = number of participants.

* = statistically significant (P-value ≤0.05).

The external responsiveness of the absolute JPE, when assessed in sitting, showed weak correlations (r range: -0.07–0.35) with measures obtained from the IMUs, while when assessed in standing, there were weak to moderate correlations (r range: 0.08–0.43). For the constant JPE, there were weak to strong correlations (r range -0.2–0.7) for the measures assessed in sitting and weak to moderate correlations (r range -0.2–0.59) when assessed in standing (Table 5).

**Table 5:** Correlation of changed scores between the laser pointer and the IMUs measured in sitting and standing for the absolute and constant JPE to evaluate the external responsiveness of the measure.

	Absolute error		Constant error	
	Sitting (r)	Standing (r)	Sitting (r)	Standing (r)
**Flexion**	-0.07	0.08	-0.20	-0.20
**Extension**	0.19	0.20	0.11	0.59
**Right rotation**	0.008	0.42	0.05	0.27
**Left rotation**	0.35	0.43	0.70	0.44

r = Person’s product-moment correlation coefficient.

## Discussion

Responsiveness is a fundamental characteristic of any outcome measure in research, mainly when the objective is to detect changes over time [45]. The ability to gauge changes in clinical status is particularly relevant in interventional studies, where researchers seek to evaluate the effectiveness of interventions. In this context, the present study utilised Cohen’s d effect size to assess the internal responsiveness of the measure of JPE, following recommendations by the COSMIN framework. Specifically, the study investigated the responsiveness of the JPE measure following a 4-week home-based proprioception training intervention for individuals with CNP. The study also assessed the correlation of change of scores obtained from the laser pointer and IMUs in order to assess the external responsiveness of this clinical measure. Internal responsiveness is defined as the ability of an outcome measure to detect clinically meaningful changes over a period of time, ensuring in accurate evaluation of treatment effectiveness [51]. External responsiveness on the other hand, evaluates the ability of an outcome measure to detect a change relative to an external criterion (i.e., gold standard) [51]. Evaluating responsiveness ensures that outcome measures capture meaningful changes. It aids in determining whether the measure appropriately captures improvements or deteriorations in health status, supporting patient-centred treatment and collaborative decision-making.

Overall, the change scores in JPE collectively suggest the clinical significance of the improvements observed in individuals with CNP. This is evident as the mean absolute JPE values after the 4-week proprioceptive training, were within the range of ≤3–4° and a range exceeding this typically signifies a deficit in neck proprioception [47]. Revel et al. [22] previously conducted a study to evaluate the beneficial effects of proprioception training for individuals with CNP. Their study reported a notable improvement of 2° in mean repositioning error after ten weeks of training. Our findings closely align with the mean repositioning error improvements of 1.75° when assessed in sitting and 1.97° when assessed in standing for absolute JPE.

Proprioception exercises require fine control of neck movements. The CNP participants in the current group carried out extensive training of neck relocation exercises as well as movement sense exercises consisting of fine neck movements as they performed tracing circles, ZZ bands and other shapes. The improvement in proprioception could be attributed to multiple mechanisms including reduced nociception, improved muscle coordination and reduced muscle inhibition although the exact mechanisms of improvement cannot be determined from the current study.

It is important to note that our intervention was shorter (4 weeks) but was administered with a higher dose of training compared to other studies. The results of the current study also explored the broader impact of the 4-week proprioception training intervention as participants showed a significant reduction in neck pain intensity, decreased disability, and decreased fear of movement following the proprioception training intervention. Revel et al. [22] and Jull et al. [12] reported similar positive trends, including reduced pain and disability levels in individuals with CNP following periods of proprioception training. The findings of the current study are also in line with a systematic review by Wilhelm et al. [52] which found that neck exercises were beneficial for reducing pain and disability regardless of exercise dosage. Similar results for people with CNP were also reported in another systematic review [53]. It should be noted however that although the change in the NDI score exceeded the minimal clinically important change, change for pain intensity (NRS) and kinesiophobia (TSK) did not, even though the changes were statistically significant. The participants enrolled in this study had mild -moderate pain intensity on average and only few had high levels of kinesiophobia which likely explains this.

For internal responsiveness, there was a large effect size for absolute JPE when assessed in sitting, particularly following active neck flexion, extension, and right rotation. The effect size for left rotation approximated a large effect size. In standing, a similar pattern emerged, with a large effect size found for the measure of absolute JPE for all movement directions. Large effect sizes were not observed for constant JPE when assessed in sitting or standing. This was expected and highlighted in previous research as the sum of the testing trials leads to an error closer to zero due to positive (overshooting) and negative (undershooting) values [25]. Besides cervical JPE, other tests can be used to evaluate the effects following a proprioception training intervention. One study assessed the effect of proprioception training on neck proprioception, using a different cervical outcome measure, the clinical cervical movement sense test (CCMST) [54]. Treleaven et al. [54] assessed the responsiveness of this cervical outcome measure after four weeks of proprioception training. Their intervention consisted of tracing a line with a laser pointer as accurately as possible or moving from one point to another. Their results showed a moderate effect size (Cohen’s d = 0.76) after training four times per week over the four-week period. In contrast, our results for internal responsiveness of the absolute JPE show larger effect sizes when tested in both sitting and standing. However, the current study found inadequate external responsiveness of the measure since only a weak correlation was observed between the measures using a laser pointer and IMUs for all movements in both tested positions, except for the constant JPE in the sitting during left rotation, which demonstrated a strong correlation. This could be due to the fact that the data collection from the laser pointer and the IMUs were not simultaneous, resulting in this weak correlation. It is worth noting that fatigue may have also influenced data collection, as prior research has indicated that fatigue can disrupt cervical proprioception [55,56] and extensive testing was carried out in this study.

### Study limitations

Performance bias is a limitation in the current study; only one therapist carried out the baseline assessment, administered the proprioception intervention, followed up with the participants, and carried out the follow up assessment. Given that this is not a clinical trial and rather was a study on the responsiveness of a measure, this was deemed acceptable. The population of participants included in our study were primarily young adults, which may affect the study’s generalisability to other age populations. The sample size was determined based on an a priori established sample size calculation and as a result, the study was sufficiently powered to address the research question. Nevertheless, future studies should aim to corroborate these findings in larger and more diverse populations. As mentioned above, data collection to assess the change of scores obtained from the laser pointer and the IMUs were not carried out simultaneously, thus might have affected the results leading to weak correlation, which may be considered as a limitation in this study.

## Conclusion

The cervical JPE test showed good internal responsiveness after 4 weeks of home-based proprioception training intervention. However, the external responsiveness in this current study was weak, and therefore, further research is recommended.

## Supporting information

S1 File Proprioception training intervention. (DOCX)

S2 File Demographic data. (XLSX)

S3 File Laser pointer data and internal responsiveness. (XLSX)

S4 File IMU data (absolute JPE). (XLSX)

S5 File IMU data (constant JPE). (XLSX)

S6 File External responsiveness. (XLSX)

S7 File Sub-analysis (absolute JPE). (XLSX)
