# Supplementary material for: Responsiveness of the cervical joint position error test to detect changes in neck proprioception following four weeks of home-based proprioceptive training
Source: PLoS One. 2024 May 10;19(5):e0303066. doi: 10.1371/journal.pone.0303066 (PMC11086922; doi:10.1371/journal.pone.0303066)
Supplement: S1 File — (DOCX) [file pone.0303066.s001.docx]

**Rehabilitation protocol**

The following exercises were to be carried out for a period of 4 weeks. Each exercise needs to be done twice a day, 3 times a week. Each exercise is to be done in a sitting and standing position. Each direction of movement is to repeated 10 times. The repositioning task will initially be performed 3 times with the eyes open and then 10 times with the eyes closed for each movement direction. The movement sense tasks will be performed 10 times to the left and 10 times to the right with the eyes opened.

**Exercise 1: Neck repositioning task**


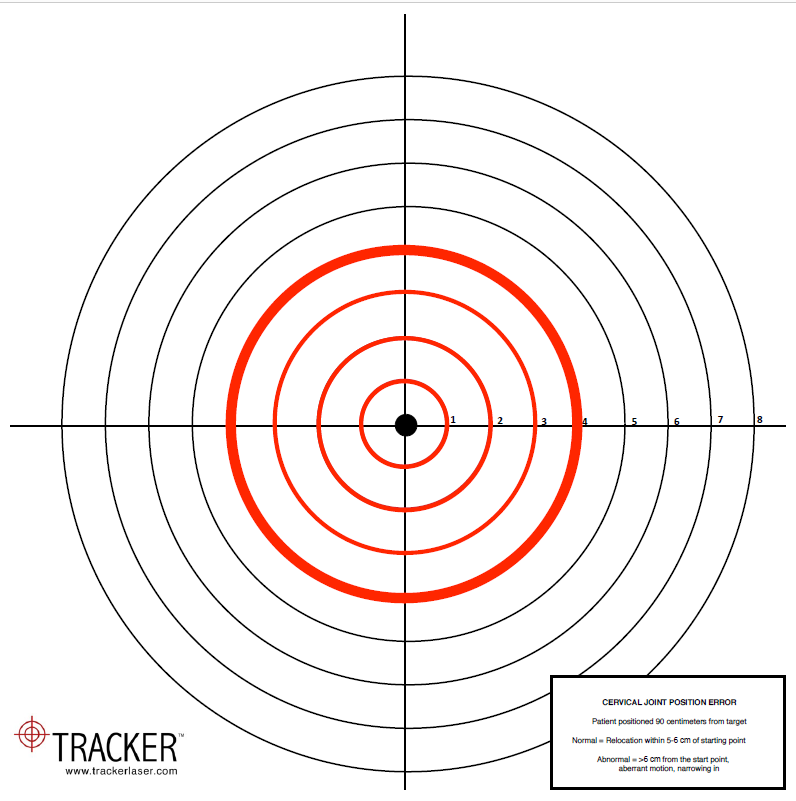


Participants will be asked to perform repetitions in flexion, extension, right and left rotation with their eyes closed. Participants will start from the bull’s eye, move to full range then return to the starting position at a comfortable pace. They will then open their eyes between repetitions and reposition themselves back to the start position. At week 2, participants will additionally perform the target head position (THP) task. They will be asked to reposition their head at mid-range in each direction.

**Exercise 2: Movement sense task (ZZ pattern)**


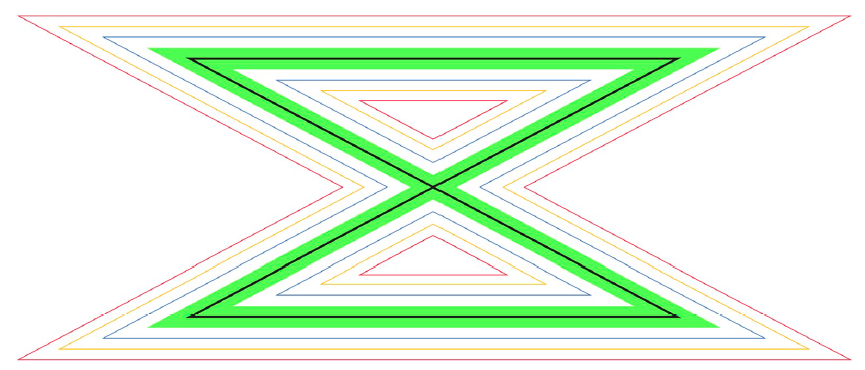


Participants will be asked to move the laser pointer in the ZZ pattern to the right and to the left to trace the pattern as accurately as possible then return to the starting point.

**Exercise 3: Movement sense task (circles with different sizes)**


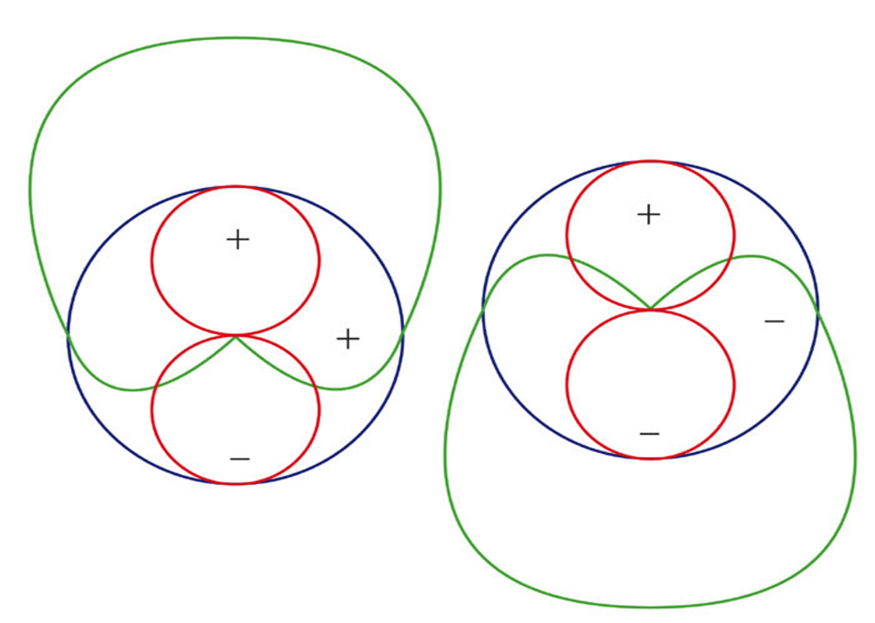


Participants will be asked to move the laser pointer to trace the lines in both clockwise and anticlockwise directions.

Week 1: Blue circle; Week 2: Green circle (right and left figures); Week 3: Red circles; Week 4 Red circles

**Exercise 4: Movement sense task**

Participants will be asked to move the laser pointer to trace the lines in both clockwise and anticlockwise directions.
